# Cellular polarity modulates drug resistance in primary colorectal cancers via orientation of the multidrug resistance protein ABCB1

**DOI:** 10.1002/path.5179

**Published:** 2019-01-16

**Authors:** Neil Ashley, Djamila Ouaret, Walter F Bodmer

**Affiliations:** ^1^ Cancer and Immunogenetics Laboratory Weatherall Institute of Molecular Medicine, University of Oxford Oxford UK; ^2^ Single Cell Genomics Facility Weatherall Institute of Molecular Medicine, University of Oxford Oxford UK

**Keywords:** primary culture, colon, spheroid, ABCB1, drug resistance, polarity

## Abstract

Colonic epithelial cells are highly polarised with a lumen‐facing apical membrane, termed the brush border, and a basal membrane in contact with the underlying extracellular matrix (ECM). This polarity is often maintained in cancer tissue in the form of neoplastic glands and has prognostic value. We compared the cellular polarity of several *ex vivo* spheroid colonic cancer cultures with their parental tumours and found that those grown as non‐attached colonies exhibited apical brush border proteins on their outer cellular membranes. Transfer of these cultures to an ECM, such as collagen, re‐established the centralised apical polarity observed *in vivo*. The multidrug resistance protein ABCB1 also became aberrantly polarised to outer colony membranes in suspension cultures, unlike cultures grown in collagen, where it was polarised to central lumens. This polarity switch was dependent on the presence of serum or selected serum components, including epidermal growth factor (EGF), transforming growth factor‐β1 (TGF‐β1) and insulin‐like growth factor‐1 (IGF‐1). The apical/basal orientation of primary cancer colon cultures cultured in collagen/serum was modulated by α2β1 integrin signalling. The polarisation of ABCB1 in colonies significantly altered drug uptake and sensitivity, as the outward polarisation of ABCB1 in suspension colonies effluxed substrates more effectively than ECM‐grown colonies with ABCB1 polarised to central lumens. Thus, serum‐free suspension colonies were more resistant to a variety of anti‐cancer drugs than ECM‐grown colonies. In conclusion, the local stroma, or absence thereof, can have profound effects on the sensitivity of colorectal cultures to drugs that are ABCB1 substrates. © 2018 The Authors. *The Journal of Pathology* published by John Wiley & Sons Ltd on behalf of Pathological Society of Great Britain and Ireland.

## Introduction

The human bowel is lined by a highly polarised cell barrier studded with small finger‐shaped pits known as crypts. Each crypt is lined by enterocytes, polarised cells with a lumen‐facing apical membrane, termed the brush border, enriched with proteins, including villin, actin and myosins 1. The opposite cell membrane in contact with the extracellular matrix (ECM) is termed the basal membrane 2. Epithelial cell polarity is orientated to the ECM via membrane proteins called integrins, consisting of protein heterodimers of α and β subunits 3, 4, 5. Colorectal cancers often retain a crypt‐like polarity in the form of neoplastic glands consisting of polarised cells surrounding a cell‐free or debris‐filled lumen 6, 7, 8 that can express polarised brush border proteins 6, 9. The formation of these neoplastic glands, or lumens, has prognostic value for the tumour and is graded routinely by pathologists 10. Well‐differentiated tumours with well‐ defined lumens have a significantly better prognosis than poorly differentiated tumours lacking lumens. The formation of polarised neoplastic glands or lumens can be modelled *in vitro* by embedding colorectal cancer cultures within three‐dimensional gels of collagen or Matrigel 11, 12, 13, 14. Within a three‐dimensional matrix, tumour colonies can form central lumens surrounded by polarised cells with an apical membrane oriented towards the lumen and a basal membrane facing the ECM, previously termed ‘apical‐in’ polarity 15. Lumen formation can be used to quantify and characterise stem cell differentiation 6, 14; in collagen gels this polarisation is modulated by integrins 12, 16, 17, 18. However, when primary cultures are grown as unattached suspension colonies in the absence of an ECM, they frequently fail to form central lumens and apical proteins like F‐actin polarise instead to membranes located at the outermost layer of the suspension colonies, facing the media 15. Similar polarity switching has been observed in other cell types 19, 20, 21 and has been termed ‘apical‐out’ polarity to distinguish it from ‘apical‐in’ polarity 15. Notably, spheroid cultures grown under ‘apical‐out’‐promoting culture conditions are frequently used in studies of cancer biology 22.

Understanding how primary cultures respond to drugs can have implications for patient drug treatments 23, 24. An abundant protein within the colon that is involved in drug sensitivity is the ABC family membrane transporter ABCB1, which acts to expel many anti‐cancer compounds from cells 25, 26, 27. Colorectal tissues express high levels of ABCB1, particularly on the apical membranes of enterocytes 28, 29, 30, 31, and ABCB1 in intestinal cancers may contribute to low efficacy of anti‐cancer drugs such as doxorubicin 32. Because ABCB1 is strongly polarised in colorectal tissues, we theorised that it would probably be influenced by the stromal and ECM interactions. To test this, we used a previously developed primary colon cancer culture system 24, as well as established cell lines, to understand the relationship between cell polarity and drug resistance. We found that ECM profoundly influenced the cellular polarity and, as a consequence, resistance to cytotoxic drugs of primary cultures.

## Materials and methods

### Sample ethics and culture conditions

Tumour samples were obtained with patient informed consent and approval by the National Research Ethics Service Committee Oxfordshire Committee C (study 07/H0606/120). All patients provided written informed consent to use tissue in research. Cultures were established as described 24. In brief, tissue was mechanically disrupted and filtered to obtain crypt‐like structures and cultured in growth factor‐enriched Excell 620 (Sigma‐Aldrich, Gillingham, Dorset, UK) serum‐free medium on low attachment plastic for 2 weeks before gradual transition to Excell 620 without growth factors. Primary cultures were transferred from these conditions for all collagen and Matrigel assays. For experiments with serum, 10% fetal bovine serum (Source BioScience, Nottingham, UK) was added to the Excell 620 medium. All cultures were tested mycoplasma free. C2284, C3953, C105251, C80 and SW1222 were genotyped using MassArray and were genetically unique from each other.

### Antibodies and reagents

Mouse anti‐carcinoembryonic antigen (CEA) was from Santa Cruz Biotechnology (Dallas, TX, USA; clone CI‐P83‐1, sc‐23928). Highly cross‐absorbed anti‐mouse Dylight 488 was from Vector Labs (Burlingame, CA, USA), highly cross‐absorbed anti‐rabbit Alexafluor 555, mouse anti‐ABCB1 (Ab‐2, clone F4), mouse anti‐ABCB1 (clone C494) and mouse anti‐ezrin (clone 3C12) were from Thermo‐Fisher (Waltham, MA, USA). Rabbit anti‐cytokeratin 20 (clone EPR1622Y), mouse anti‐villin (clone 1D2C3), rabbit anti‐villin (SP145) and rabbit anti‐ABCB1 (EPR10364‐57) were from Abcam (Cambridge, UK). Rabbit anti‐β actin (13E5) and anti‐mysosin IIC were from Cell Signaling Technology (Danvers, MA, USA). The rat anti‐integrin β1 chain (clone AIIB2) and rat anti‐integrin α5 chain (clone BIIG2) 33 were obtained from the Developmental Studies Hybridoma Bank (University of Iowa, Iowa City, IA, USA). Anti‐integrin β1 chain (clone P5D2) and anti‐integrin α1β2 (clone BHA2.1) were from Millipore (Watford, UK). 4′,6‐diamidino‐2‐phenylindole (DAPI), blebbistatin, nocodazole, cytochalasin B, phalloidin–tetramethylrhodamine B isothiocyanate (TRITC; used to label F‐actin), tetramethylrhodamine, ethyl ester (TMRE), Matrigel (growth factor reduced) were from Sigma‐Aldrich. PrestoBlue/SytoxBlue were from Thermo‐Fisher and obtustatin was from R&D Systems (Minneapolis, MN, USA). All ABCB1 immunostaining was validated with three different anti‐ABCB1 clones detailed above. Cetuximab anti‐epidermal growth factor receptor (EGFR) monoclonal antibody (20 μg/ml) was from Millipore, transforming growth factor‐β (TGF‐β; 10 ng/ml) was from PeproTech EC (London, UK), EGF, recombinant (10 ng/ml) was from Lonza (Burton on Trent, UK), insulin‐like growth factor‐1 (IGF‐1; 1 μg/ml) was from Thermo‐Fisher. All antibodies were used at 1:100 dilution, except secondary antibodies (1:800).

### Immunofluorescence, microscopy and imaging

Haematoxylin/eosin and immunofluorescence labelling of whole colonies or formalin‐fixed paraffin‐embedded (FFPE) sections was carried out as described previously 6. Live cell imaging was performed using a Zeiss Axio Observer microscope in a humidified and CO_2_ supplemented chamber at 37 °C. Confocal imaging of whole mounted colonies and FFPE sections was performed using a Zeiss LSM 510 laser scanning microscope. For ABCB1 fluorescence, FIJI software was used to measure the immunofluorescence of ABCB1, using the mean grey values of each colony.

### Collagen gel/Matrigel assays

Bovine collagen I (600 μl, Life Technologies, Warrington, UK) was mixed with 275 μl water and 100 μl 10× RPMI1640 medium (Thermo Fisher). The pH was neutralised by the addition of 20–25 μl NaOH (until the colour became pink). Colonies were purified using a 40 μm nylon mesh (Fisher Scientific) and mixed with the collagen. Mixes (10 μl) were added to the lower wells of Ibidi angiogenesis slides (Ibidi, Munich, Germany). Antibodies, growth factors or small chemical inhibitors were mixed into the gel at this point (no more than 1:5 vol:vol). Antibodies were added to the gel at 8–10 μg/ml. The gels were allowed to set at 37 °C for 60 min and overlaid with 60 μl medium. Excell 620 medium was replaced every 48 h.

For Matrigel assays, colonies were filtered as above and mixed with a 50:50 mix of Matrigel/Excel medium in Ibidi angiogenesis slides 6.

### Viability assays

Microplate‐based viability assays using PrestoBlue/SytoxBlue were performed on filter‐purified colonies, as described previously 24. Values were normalised using parallel detergent‐treated cultures. Direct counting of dead cells in colonies was performed using SytoxBlue. Colonies in collagen gels were cultured for 4 days and then fresh medium containing the agent to be tested was added and was replaced every 48 h for 4 days. Spent medium was then replaced by fresh prewarmed medium containing 1 μm SytoxBlue and incubated at 37 °C for 30 min. A phase‐contrast/fluorescence image stack (*x* = 3581.04 μm, *y* = 2683.2 μm) through the middle of each gel was acquired using a 20× objective. Each phase‐contrast image was processed manually to outline colonies in focus using the ROI manager tool in Fiji imaging software. SytoxBlue foci per colony were then automatically counted using the approach used previously to count F‐actin foci 6, except that edge exclusion was not used.

### ABCB1 function assays using Rhodamine 123 or TMRE

For Rhodamine 123 (R123) or TMRE uptake assays, primaries were filter purified and either placed in fresh Excell 620 or embedded in collagen/Matrigel and cultured for 4 days. Medium was replaced by fresh prewarmed medium containing 1 μm R123 or 100 nm TMRE for 30–60 min. For R123 labelling the colonies were washed twice with 60 μl fresh medium with no R123.

### Real‐time imaging of primary cancer cultures and image analysis

Colonies were embedded in Matrigel/collagen gels as described above and cultured with Excell 620 in a 96‐well plate. Primary cultures were imaged using the oCelloscope microscope scanning system (Philips Biocell, Frederiksborg, Denmark), as described previously 24.

### Microarray analysis

Gene microarray expression analyses were performed using the Affymetrix Human Genome U133+2 chips. Total RNA from ∼100 primary colonies (C3953, C2284, C105251, C4054) was extracted using the RNeasy kit (Qiagen, Manchester, UK), converted to cDNA using the high‐capacity RNA‐to‐cDNA Kit (Thermo‐Fisher). All samples were processed in accordance with the Affymetrix protocol and 2 μg fragmented and labelled cDNA was hybridised to the Affymetrix GeneChip U133+2 arrays. Partek Genomic Suite software was used to compare expression profiles of primary cultures. For genes with significant differential expression, step‐up correction for multiple testing was used (with a Benjamini and Hochberg step‐up false discovery rate‐corrected *P* value cut‐off of 0.05). Microarray data are available in supplementary material, Table S1.

## Results

We established *ex vivo* primary cultures from resected patient colorectal tumours as described in detail previously 6, 24 using non‐attachment plastic culture vessels and serum‐free medium. These cultures grow as unattached spheroid colonies. Haematoxylin/eosin staining of the parental tumours indicated a well or moderately differentiated phenotype, as defined by the presence of neoplastic glands (see supplementary material, Figure S1A). Immunostaining for the brush border proteins actin, ezrin, villin, myosin IIC (MYH14) and CEA 13, 24, 34, 35 showed polarised brush border protein expression at the apical membranes surrounding the lumens, with some polarisation also at the basal membranes (see supplementary material, Figure S1B–D). Consistent with the report by Okuyama *et al* 15, we found that in corresponding daughter spheroid cultures, brush border proteins were strongly polarised to outer cell membranes facing the medium, thus inverted from their *in vivo* orientation, which has been termed ‘apical‐out’ polarity. Rare lumens occasionally present in these suspension cultures also expressed polarised brush border markers.

**Figure 1:**
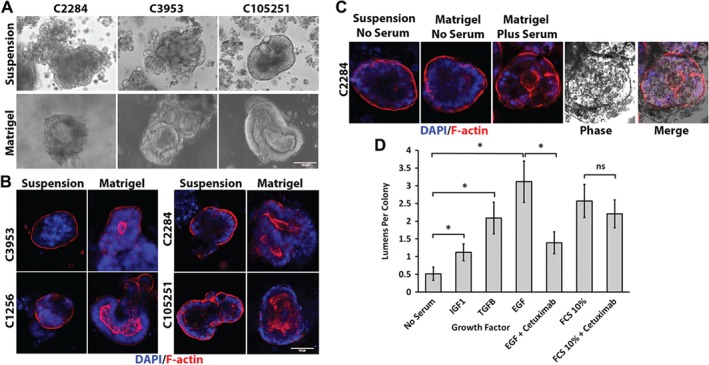
Lumen formation and restoration of *in vivo* polarity of primary cultures in ECM depends on the presence of serum or the growth factors EGF, TGF‐β1 and IGF‐1. (A) Phase‐contrast images of three primary colorectal cancer cultures cultured for 1 week as either serum‐free suspension cultures (upper panels) or embedded in Matrigel with added serum (lower panels). (B) F‐actin (red)/DAPI (blue) staining of colorectal cancer cultures from four patients cultured as a serum‐free suspension or in Matrigel with serum for 1 week. (C) F‐actin (red)/DAPI (blue) labelling of a C2284 primary culture grown as either a serum‐free suspension (left two panels) or in Matrigel with or without serum added. Phase image is included to demonstrate F‐actin‐lined lumen. (D) Effect of various serum growth factors on lumen formation in primary cultures cultured in Matrigel. C2284 primary serum‐free suspension cultures were embedded in Matrigel and the serum‐free medium was supplemented with either TGF‐1β (10 ng/ml), EGF (10 ng/ml) or IGF‐1 (1 μg/ml). Cetuximab anti‐EGFR monoclonal antibody was used at 20 μg/ml. Lumen formation was measured by F‐actin labelling. Ten per cent serum and no serum medium controls were used as positive and negative controls for lumens, respectively. At least 30 colonies were counted per condition (*n* = 3). Error bar = standard error of the mean. *p < 0.05 paired *t‐*test. Paired *t‐*test of 10% serum + cetuximab was not significantly different from the 10% serum control.

Primary cultures can form lumens enriched with the polarity/brush border marker F‐actin when embedded in Matrigel, an ECM containing collagens 6. We utilised this approach to determine whether normal apical polarity could be restored in primary spheroid cultures when cultured within Matrigel with or without serum media, compared with parallel cultures maintained as serum‐free suspension spheroid colonies. When grown in Matrigel in the presence of serum, virtually all the primary colonies formed central lumens (Figure 1A), which were strongly labelled by polarised F‐actin (Figure 1B, supplementary material, Movie S1). Lumen formation was consistent with the pathological grading of the cultures' parental tissues as moderate or well differentiated. The same cultures maintained as colony suspensions without serum or Matrigel did not form lumens and showed a marked apical‐out polarity of F‐actin. Other brush border/polarity markers, actin/ezrin, myosin IIC/villin, also partly relocated from the apical‐out to apical‐in polarity of central lumen‐facing cell membranes when embedded in Matrigel (see supplementary material, Figure S2A,B). This polarity switch was dependent on serum as colonies embedded in Matrigel without serum retained apical‐out F‐actin expression and did not form lumens (Figure 1C).

We investigated the factors present in serum that could contribute to this polarity switch by adding recombinant growth factors to primary cultures suspended in Matrigel but maintained in serum‐free media (Figure 1D). Colonies without added growth factors formed only low levels of lumens, but the number of lumens was significantly increased in cultures with added EGF, TGF‐β or IGF‐1. The effect of EGF on lumens was mitigated by the anti‐EGFR monoclonal antibody inhibitor cetuximab, but cetuximab did not significantly inhibit lumen formation induced by serum, suggesting that the effects of serum were due to factors other than only EGF.

**Figure 2:**
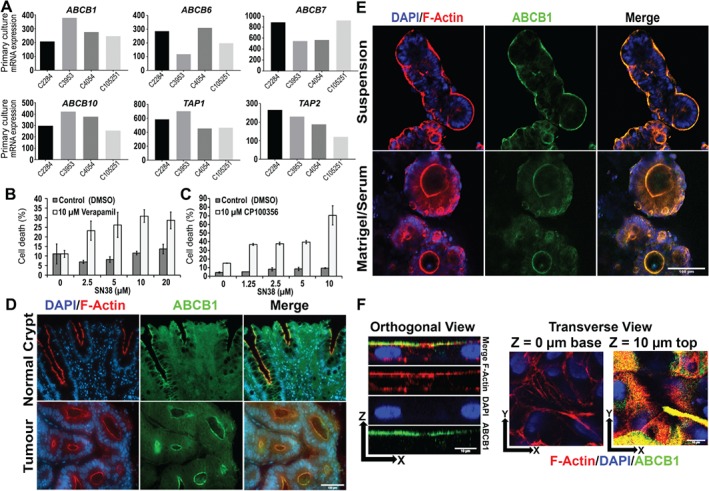
ABCB1 is polarised to apical‐out and apical‐in orientations in suspension or ECM conditions, respectively, and is involved in resistance to cytotoxic drugs. (A) mRNA levels of ABC family transporters in four primary cultures, determined by microarray analysis. (B) Cell death in serum‐free suspension colonies (C105251) exposed to various concentrations of SN38 in the presence or absence of ABCB1 inhibitor verapamil (10 μm) or vehicle control (DMSO). (C) Same experiment as (B) except using the alternative ABCB1 inhibitor CP100356 (10 μm) compared with vehicle controls (DMSO). The SytoxBlue versus PrestoBlue fluorescence was normalised to detergent‐treated controls (see Materials and methods). *p ≤ 0.05 verapamil versus control (Student's *t*‐test). Error bars = standard error of the mean. (D) F‐actin (red)/DAPI (blue) and anti‐ABCB1 (green) labelling of cross‐sections of normal colonic crypt tissue and well‐differentiated colonic tumour. Scale bar = 100 μm. (E) Co‐labelling for F‐actin (red), ABCB1 (green) with DAPI (blue) in C105251 primary cultures grown as either serum‐free suspension spheroids or as Matrigel‐embedded cultures in the presence of serum. Scale bar = 100 μm. (F) Orthogonal and transverse views of co‐labelling for ABCB1 (green) and F‐actin (red) with DAPI (blue) in a C105251 primary culture grown as a monolayer for 2 weeks in the presence of serum on cell culture‐treated plastic. Scale bar = 10 μm.

Because ABCB1 is enriched in the intestinal brush border 28 it might be influenced by polarity switching *in vitro*. Microarray analysis of four primary cultures confirmed that they expressed a number of ABC transporters, including ABCB1 (Figure 2A). We confirmed that ABCB1 was active in the primary colonies by measuring cell death due to exposure to the irinotecan metabolite SN38 in the presence or absence of the ABCB1 inhibitors verapamil and CP‐100356. The ABCB1 inhibitors significantly increased the sensitivity of C105251 and C3953 serum‐free/suspension cultures to SN38‐mediated cell death (Figure 2B,C, respectively). We next examined the tissue distribution of ABCB1 using immunolabelling. In human colonic tissue sections, ABCB1 was highly polarised to apical brush border membranes in normal colonic crypts and also in the neoplastic glands of differentiated colorectal adenomas, colocalising with F‐actin (Figure 2D). *In vitro* cultures showed that ABCB1 colocalised with F‐actin in an apical‐out orientation when grown as serum‐free suspensions and an apical‐in orientation when grown in Matrigel with serum (Figure 2E, supplementary material, Figure S3). A similar apical‐out/apical‐in polarisation of ABCB1 to that seen for the primary cultures was observed in suspension‐ versus Matrigel‐grown colonies of the established colorectal cell lines C80 and SW1222 (see supplementary material, Figure S4), although SW1222 cell colonies did not fully relocate ABCB1 to the outer membranes when grown as serum‐free spheroids.

ABCB1 was also strongly polarised to the apical membranes facing the medium of primary cultures grown as conventional cell monolayers in the presence of serum, presumably because of ECM components present in the serum 24 (Figure 2F). Supplementary material, Movie S2 shows a three‐dimensional reconstruction of ABCB1 (green) in the monolayer.

**Figure 3:**
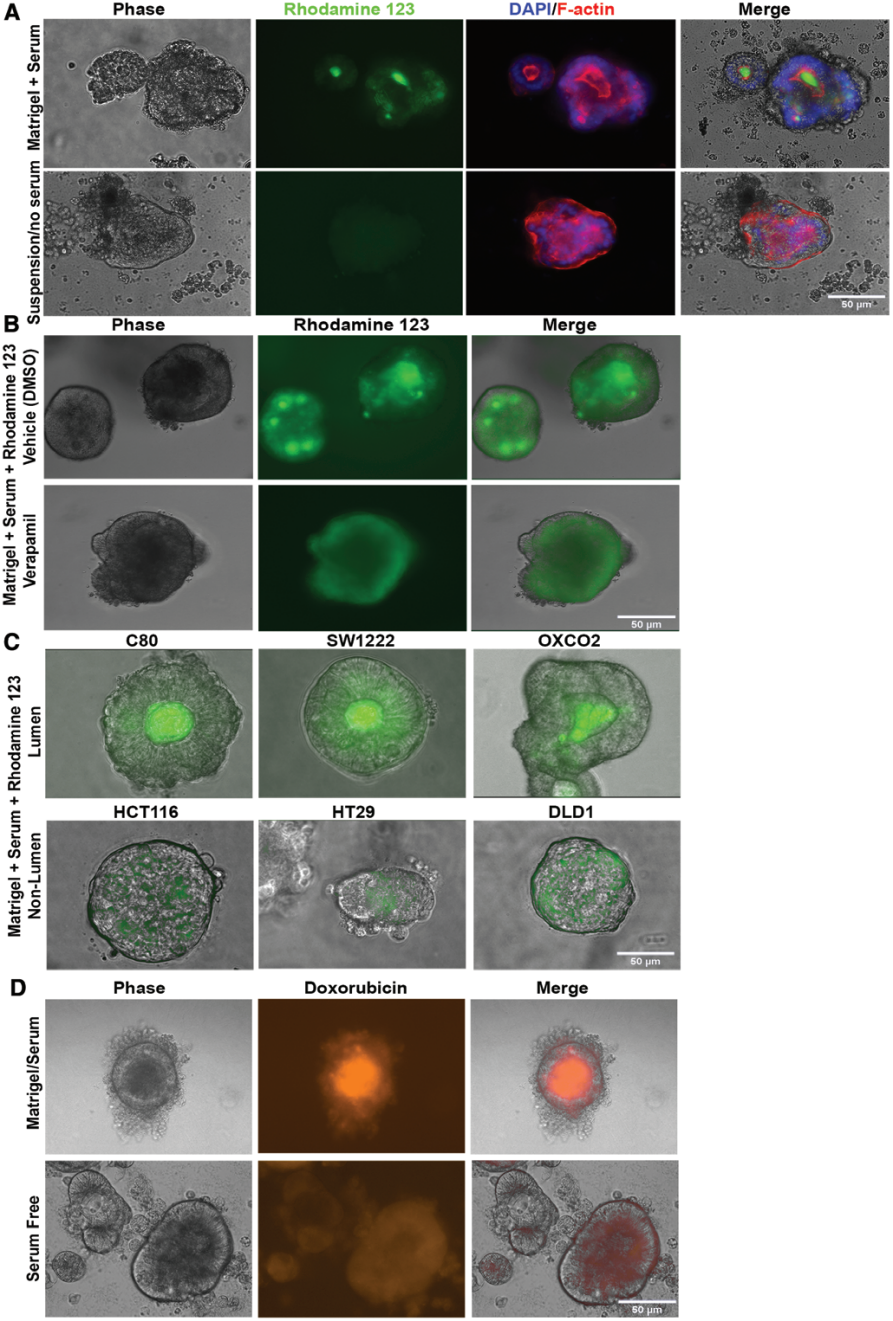
Polarised ABCB1 functions as an orientation‐dependent drug effluxer. (A) C3953 colonies cultured in either Matrigel/serum or as serum‐free suspensions for 1 week and labelled with the fluorescent ABCB1 substrate R123 (green) for 1 h. The same colonies were then fixed and labelled for F‐actin (red) with DAPI (blue). Merge shows R123/DAPI/phase contrast/F‐actin. (B) R123 fluorescence of C3953 colonies grown in Matrigel/serum for 1 week in the presence of either DMSO (vehicle) or 50 μm verapamil. (C) R123 labelling of live Matrigel/serum‐grown lumen‐forming colorectal cell lines (C80, SW1222, OXCO2) and non‐lumen‐forming cell lines (HCT116, HT29 and DLD1). (D) Doxorubicin auto‐fluorescence of live C105251 colonies cultured in either Matrigel/serum for 1 week or as serum‐free suspension cultures and incubated with 10 μm doxorubicin for 1 day. Scale bars = 50 μm.

To determine how the apical‐out and apical‐in polarisation of ABCB1 affected accumulation of its substrates we used the fluorescent ABCB1 substrate R123 36 to monitor ABCB1 substrate accumulation in C3953 colonies cultured either in Matrigel with serum or in suspension without serum. Figure 3A (upper panels) shows that R123 accumulated in distinct fluorescent foci within the colonies cultured with Matrigel and serum and these fluorescent foci were coincident with lumens observable by phase contrast or by F‐actin labelling. By contrast there was very little R123 fluorescence in the serum‐free suspension colonies, which also lacked F‐actin/phase contrast‐labelled lumens (Figure 3A, lower panels). To confirm that the intracellular accumulation of R123 in lumens in serum‐grown colonies in Matrigel was due to the activity of ABCB1, we incubated lumen‐containing colonies grown in Matrigel/serum with R123 in the presence of either the ABCB1 inhibitor verapamil or the vehicle control. Figure 3B shows that verapamil markedly reduced the accumulation of R123 foci within the lumen colonies compared with the vehicle control, supporting the concept that apical‐in ABCB1 actively pumped R123 into lumens where it accumulated as brightly fluorescent foci.

We have previously reported that some established colon cancer cell lines, such as SW1222, form lumens when embedded in Matrigel, whereas others, such as HT29, do not 14. We compared the uptake of R123 by lumen‐ and non‐lumen‐forming cell lines embedded in Matrigel/serum. R123 accumulated strongly within the lumens of lumen‐forming C80, OXCO2 and SW1222 cell line colonies grown in Matrigel/serum, but not in non‐lumen‐forming cell lines HCT116, HT29 and DLD1 (Figure 3C). In addition, C80 cell colonies grown in Matrigel/serum also accumulated the red fluorescent ABCB1 substrate TMRE 37 in lumens, whereas C80 grown as serum‐free suspensions showed a weaker, more diffuse TMRE fluorescence (see supplementary material, Figure S5, upper two panels). Furthermore, verapamil, and the more specific ABCB1 inhibitor CP‐100356, both inhibited the accumulation of TMRE in C80 Matrigel/serum lumens (see supplementary material, Figure S5, lower two panels). The fluorescent anti‐cancer drug doxorubicin was also observed to accumulate in the lumens of Matrigel‐cultured primary C105251 colonies, but not serum‐free suspension colonies (Figure 3D). There was no significant difference in the ABCB1 expression level, as measured by immune fluorescence, between Matrigel/serum and serum‐free suspension colonies (see supplementary material, Figure S6A). Collectively these results indicate that the apical‐in orientation of ABCB1 in ECM/serum colonies leads to accumulation of ABCB1 substrates within lumens, whereas the apical‐out orientation of ABCB1 in suspension colonies excludes ABCB1 substrates.

**Figure 4:**
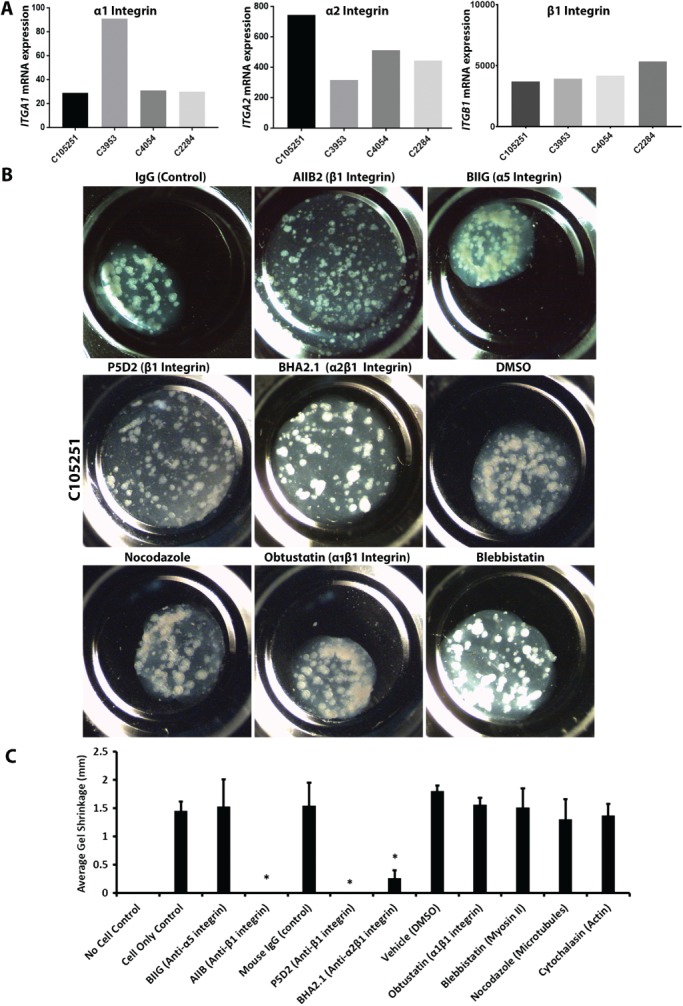
Integrin α2β1 mediates the interaction of primary cultures with collagen. (A) Microarray gene expression analysis of various integrin subunits in the primary cultures. (B) Phase‐contrast images of collagen I gels in which C105251 primary cultures were incubated with various inhibitors or controls for 3 days. These include isotype control mouse monoclonal IgG, AIIB2 (rat monoclonal against integrin β1 subunit), BIIG (control rat monoclonal against integrin α5 subunit), P5D2 (mouse monoclonal against integrin β1 subunit), BHA2.1 (mouse monoclonal against α2β1 integrin heterodimer). Each antibody was used at 10 μg/ml. Other inhibitors tested included obtustatin (α1β1 inhibitor, 5 nm), blebbistatin (myosin II inhibitor, 100 μm). (C) Graph showing quantification of C105251‐mediated gel contraction, of the same experiment as (B) with the additional inhibitors nocodazole and cytochalasin B (microtubule and actin inhibitors, respectively, 1 μm each). DMSO was the vehicle control. *p < 0.05 versus appropriate control, Dunnett's multiple comparisons test. Error bar = standard deviation.

β1 integrin is important for lumen formation in colonies of established colon cancer cell lines in collagen gels 17, 38, 39 and our microarray analysis showed that the primaries expressed RNA for β1, α1 and α2 integrin subunits (protein expression was not measured) (Figure 4A). We therefore investigated the role of β1 integrin/collagen interactions in ABCB1 polarisation by embedding the primary cultures in collagen I gels with serum. Unexpectedly, within a period of 24–72 h, three of four primary cultures markedly shrank the collagen gels they were embedded in (see example shown in supplementary material, Movie S3). The primaries mechanically distorted the local collagen and collagen fibrils were pulled towards the colony. Quantitative measurement of this collagen gel contraction by primary cultures enabled us to study the interaction of the colonies with collagen. We embedded equal numbers of colonies in a fixed volume of collagen gel layered into microwells of uniform dimensions. The resulting gels initially had a uniform width, which could then be measured over 3 days to determine the extent of gel contraction quantitatively. Using this approach, we measured the effect of various collagen‐related inhibitors and function‐blocking antibodies on C105251‐mediated collagen contraction (Figure 4B,C). Function‐blocking antibodies AIIB and P5D2 that target the integrin β1 subunit 40, 41 markedly inhibited gel contraction, as did a blocking antibody reported to be specific for the α2β1 integrin heterodimer. Mouse and rat isotype control antibodies (IgG and BIIG2, respectively) did not significantly inhibit gel contraction, and gels with no cells did not shrink. Obtustatin, a potent inhibitor of α1β1 integrin 42, also had no effect on gel contraction. These results suggest that α2β1 integrin is the main receptor for collagen I for primary colon cancer cultures. In addition, there was no reduction in gel contraction with inhibitors to actin polymerisation (inhibited by cytochalasin B), microtubules (inhibited by nocodazole) or myosin II (inhibited by blebbistatin). Thus, the interaction of primaries with collagen I is primarily mediated via α2β1 integrin and does not involve α1β1 integrin, actin, myosin II or microtubule function. The same cultures embedded in Matrigel did not shrink the gel, probably as it is softer than collagen.

**Figure 5:**
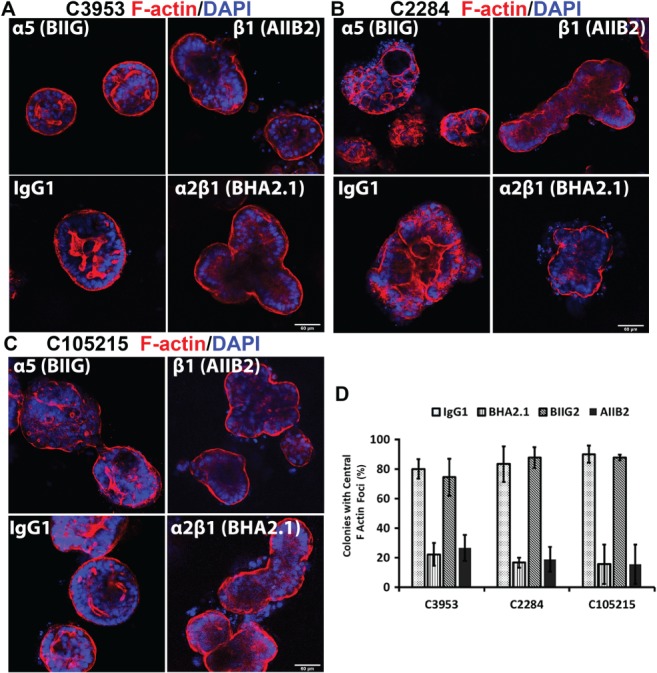
β1 and α2β1 integrin function‐blocking antibodies prevent switching of apical‐out polarity to apical‐in polarity in primary cultures grown in collagen I gels with serum. (A–C) F‐actin/DAPI labelling of C3953, C2284 and C105251 cultures grown for 5 days in collagen I/serum gels with either isotype control antibodies (IgG or BIIG) or the β1 integrin‐blocking antibody AIIB2 or α1β2‐blocking antibody BHA2.1. (D) Quantification of F‐actin foci larger than 3 μm of the same experiment as presented in (A–C). Thirty colonies for each condition were counted (three experiments). Antibodies were used at 10 μg/ml each.

We tested whether the blockade of α2β1 inhibited apical polarisation of primary cultures by using F‐actin as a marker of polarity. Figure 5A–C shows various primary colonies cultured for 5 days in collagen I/serum with either isotype controls (IgG and BIIG) or with β1/α2β1‐inhibiting antibodies (AIIB and BHA2.1, respectively). Cultures grown with control IgG predominantly showed apical‐in polarity with internal lumens marked by F‐actin. By contrast, colonies grown in the presence of the β1 or α2β1 function‐blocking IgGs showed a predominantly apical‐out polarity. Quantification of the F‐actin foci in the same cultures grown in collagen I/serum gels confirmed a significant inhibition of central lumens in colonies cultured in the presence of β1 or α2β1 function‐blocking IgGs (Figure 5D). These data suggest that α2β1 integrin is the main collagen ligand that establishes polarity orientation in primary cultures when cultured in type collagen I gels.

**Figure 6:**
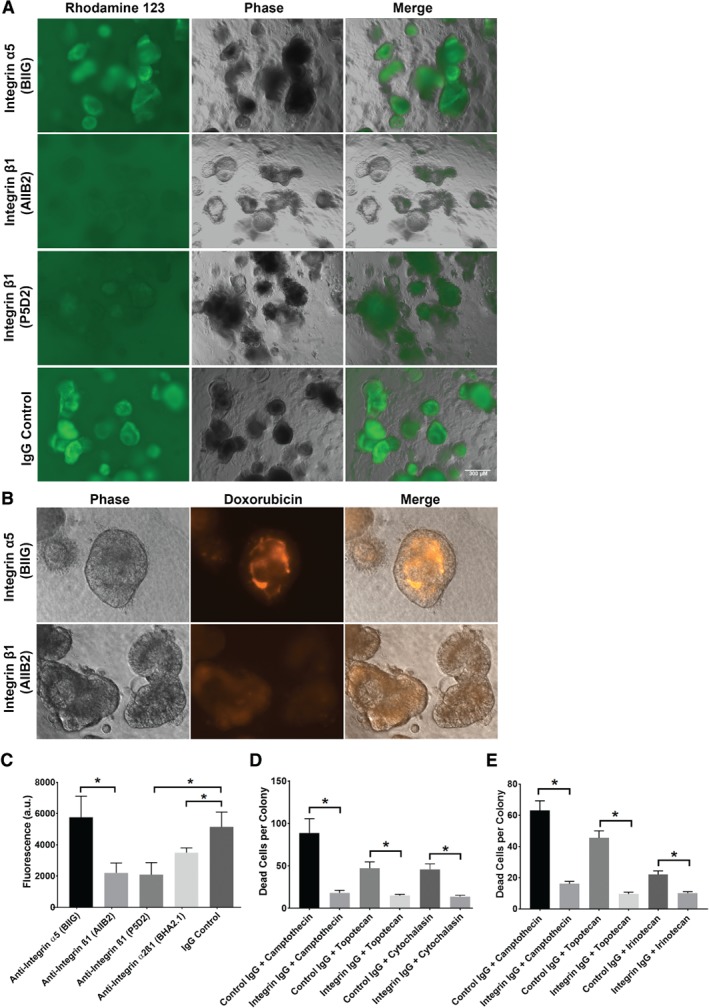
β1 integrin inhibition of collagen/serum‐grown primary cultures reduces their uptake of ABCB1 substrates and decreases their drug sensitivity. (A) R123 labelling (1 mm, 1 h) of live C105251 cultures grown in collagen with serum for 5 days in the presence of either isotype control antibodies (mouse IgG or rat anti‐integrin α5 [BIIG] IgG) or β1 integrin‐blocking antibodies (AIIB2 or P5D2 IgG, 10 μg/ml each). (B) Doxorubicin (10 μm for 6 h) fluorescence of live C105251 cultures grown in collagen with serum for 5 days in the presence of either control antibodies (BIIG) or β1 integrin function‐blocking antibody (AIIB2). (C) Plate reader measurement of R123 fluorescence in C105251 from the experiment presented in (A). AIIB2‐, P5D2‐ and BHA2.1‐treated cultures showed significantly reduced R123 fluorescence compared with controls (*p* = 0.015, *p* = 0.013, *p* = 0.04, respectively, Student's *t*‐test). (D) Cell death (normalised to vehicle/IgG controls) in C105251 cultured in collagen with either control antibodies (IgG/BIIG) or β1 integrin‐blocking antibodies (AIIB2 IgG/P5D2 IgG), in the presence of various chemotherapy drugs: camptothecin (20 μm), topotecan (10 μm), cytochalasin B (5 μm). Cell death was measured *in situ* in each colony by counting the number of SytoxBlue foci (a dye specifically staining dead cells), using a FIJI algorithm of digital images, as detailed in Materials and methods (*n* = 30–50). (E) Identical experiment to (D) but using C2284 primary cultures and irinotecan (10 μm) instead of cytochalasin B. Antibodies were used at 10 μg/ml each. Error bars = standard error of the mean. *p < 0.05 drug/control IgG versus drug/β1 integrin IgG.

Inhibition of β1 integrin signalling in collagen‐grown cultures also prevented ABCB1 from relocating from an apical‐out to an apical‐in orientation (see supplementary material, Figure S6B) and they maintained high ABCB1 in the outer layer of cells facing the media. We hypothesised that apical‐out ABCB1 would increase the efflux of ABCB1 substrates from colonies, decreasing drug sensitivity. We tested this with R123 labelling of primary cultures incubated with control or β1 integrin‐blocking antibodies in collagen/serum gels. Figure 6A shows that β1 integrin‐blocking antibodies reduced the uptake of R123 by C105251 colonies grown in collagen/serum for 5 days, compared with cultures with control antibodies. We measured this quantitatively using a fluorescent plate reader (Figure 6C), confirming a significant decrease in fluorescence for β1 and α2β1 integrin‐blocking antibodies compared with the isotype controls. Blockade of β1 integrin also reduced the internal accumulation of doxorubicin in C2284 colonies cultured in collagen gel with serum, compared with the control (Figure 6B). We also measured drug‐induced cell death in C105251 and C2284 colonies grown in collagen/serum gels with either control or integrin‐blocking IgGs (Figure 6D,E). Integrin inhibition significantly reduced cell death in these cultures when exposed to camptothecin, topotecan, irinotecan and cytochalasin B, compared with cultures incubated with the control IgG. Thus, the efflux of drugs via apical‐out polarised ABCB1 is capable of protecting cancer cells from cell death due to cytotoxic drugs.

## Discussion

The main finding of our study is that ABCB1, in common with other intestinal brush border components, is a polarised protein whose cellular location is influenced by the surrounding ECM. Thus, in serum‐free primary suspension cultures that have undergone an ‘inside‐out’ polarity switch due to a lack of ECM signalling, the outer cells of the colony mass facing the medium become enriched with ABCB1 in their outer membranes (‘apical‐out’ orientation). Similar apical‐out polarisation of ABCB1 was also observed in primary cultures grown on conventional tissue culture plastic, presumably due to ECM components in the serum.

By contrast, for *in vivo* tissue, the main orientation of ABCB1 is apical‐in, with enrichment at central lumen‐facing cell membranes, both in normal colon crypts and in neoplastic tumour glands. This apical‐in orientation of ABCB1 could be reproduced *in vitro* by reintroducing an ECM, such as Matrigel, to the suspension colonies in a serum‐dependent manner. ECM‐embedded colonies without serum retained apical‐out polarity. The known serum growth factors EGF, IGF‐1 and TGF‐β could individually promote lumen formation in our primary cultures, indicating that multiple factors within serum may collectively contribute to promote ECM signalling.

Integrins were important for this apical‐out to apical‐in transition in ECM, as α2β1 integrin function blocking prevented lumen formation of primary suspension cultures transferred to collagen gels. These colonies also retained the apical‐out polarity of suspension cultures. This is consistent with previous reports that β1 integrin blockade can inhibit lumen formation by colorectal cancer cell lines cultured in collagen 15, 38, 39. Function blocking of α2β1 integrin, but not α1β1 integrin, also inhibited the ability of primary cultures to shrink the collagen gel in which they were embedded, suggesting that α2β1 rather than α1β1 is the main collagen I receptor on the primary cultured cells. Collagen contraction, a property only previously reported for certain cultured intestinal epithelial cell lines 43, 44, was also unaffected by inhibitors of actin, myosin II or microtubules.

In apical‐in primary cultures grown in ECM/serum, fluorescent ABCB1 substrates and doxorubicin accumulated strongly in lumens, whereas apical‐out colonies accumulated much less of these markers. Matrigel/serum‐embedded non‐lumen‐forming cell line colonies also accumulated less ABCB1 substrates than lumen‐forming cell line colonies. Similar luminal accumulation of ABCB1 substrates has been reported in mouse intestinal organoids grown in Matrigel 36. When apical‐out polarity was maintained in colonies cultured in collagen/serum by using a β1 integrin blockade, the colonies accumulated less rhodamine/doxorubicin and were less sensitive to cytotoxic ABCB1 substrates than apical‐in colonies grown under the same conditions. This indicates that cellular polarity, rather than differences in culture conditions, dictates the accumulation of ABCB1 substrates within the colonies. In either apical‐in or apical‐out colonies, ABCB1 substrates would still be expelled from the cells by ABCB1 but the key difference is where the substrates are expelled to. For apical‐in colonies, ABCB1 substrates would be able to enter the entire cell mass of each colony from the outside before expulsion by apical ABCB1 into the central lumens. In the case of substrates that were cytotoxic, such increased exposure would increase the toxicity within the colony mass. By contrast, for apical‐out colonies, the ABCB1 substrates mainly enter the outer layer of cells where the high level of outwardly orientated ABCB1 effectively forms a protective barrier that excludes ABCB1 substrates, such as doxorubicin, from the inner colony cell mass. Inner colony cells are therefore exposed to lower concentrations of cytotoxic ABCB1 substrates. Thus, cytotoxic ABCB1 substrates have a reduced effect on cells within the apical‐out colonies compared with apical‐in colonies. Although integrin signalling can increase drug resistance under certain culture conditions 45, this is context dependent, as the loss of integrin signalling in ECM‐embedded three‐dimensional breast cancer colonies can increase drug resistance by restoring colony polarisation 46.

Although apical‐out polarity switches have been reported before 15, 20, 21, 47, our study is the first to implicate drug effluxers in this process. As primary cultures express several other ABC family members, it is possible that other membrane transporters are influenced by cellular polarity. Polarity switching probably accounts for the layer of irinotecan‐resistant ABCB1/cytokeratin 20 high cells previously described in serum‐free colorectal spheroids 48. However, it is clear that for the differentiated tumours we examined in this study, the apical‐out expression of brush border proteins, such as ABCB1, does not reflect their *in vivo* polarity. ECM cultures should therefore be preferred over suspension spheroid or plastic‐grown cultures, particularly for drug sensitivity studies.

In conclusion, we have shown that polarity orientation is an important factor for primary colorectal cancer cultures *in vitro*, and that this should be taken into account when interpreting results from experimental systems that lack appropriate ECM signalling.

## Author contributions statement

NA, DJ and WFB conceived the study design. NA and DJ performed the experiments. NA and WFB wrote the report.

**SUPPLEMENTARY MATERIAL ONLINE**

**Supplementary figure and movie legends**

**Figure S1.** Immunostaining for polarity markers actin, ezrin, villin and myosin IIc, in six parental tumours and daughter primary serum‐free suspension cultures

**Figure S2.** Apical‐in orientation of brush border proteins is restored in primary cultures grown in Matrigel with serum

**Figure S3.** ABCB1 is polarised to outer colony cell membranes in serum‐free suspension but relocates to central apical membranes in cultures grown in Matrigel/serum

**Figure S4.** Established colorectal cancer cell lines have polarised ABCB1

**Figure S5.** C80 colonies grown in Matrigel/serum accumulate the ABCB1 substrate TMRE in lumens in an ABCB1‐dependent manner

**Figure S6.** ABCB1 distribution in colonies under different culture conditions

**Movie S1.** Z‐stack of red F‐actin and blue DAPI staining of primary culture C2661 cultured in Matrigel with 10% serum for 1 week

**Movie S2.** Three‐dimensional reconstruction of a C105251 monolayer grown on plastic in serum‐containing medium for 2 weeks, labelled for ABCB1 (green), F‐actin (red) and with DAPI (blue)

**Movie S3.** Primary cultures have collagen‐contracting capacity

**CEL files.** Four CEL files each with raw microarray data from a cell line

**Table S1.** Microarray data used in Figures 2 and 4

## Supporting information


**Supplementary figure and movie legends**

**Figure S1. Immunostaining for polarity markers actin, ezrin, villin and myosin IIc, in six parental tumours and daughter primary serum‐free suspension cultures.** (A) Haematoxylin/eosin staining of parental tumour sections. (B) Anti‐actin (red)/anti‐ezrin (green)/DAPI co‐staining or (C) anti‐villin (green)/anti‐myosin IIC (red)/DAPI (blue) of parental tumour sections and corresponding daughter suspension cultures. (D) Anti‐CEA labelling in green, DAPI in blue. Bar = 50 μm.

**Figure S2. Apical‐in orientation of brush border proteins is restored in primary cultures grown in Matrigel with serum.** Primary suspension cultures were cultured as either serum‐free suspensions or serum‐containing Matrigel cultures for 1 week, followed by fixation, sectioning and immunolabelling. (A) Anti‐actin (red)/DAPI (blue) and anti‐ezrin (green) labelling of suspension/Matrigel cultures, with phase‐contrast images. (B) Anti‐myosin II (red)/DAPI (blue) and anti‐villin (green) labelling.

**Figure S3. ABCB1 is polarised to outer colony cell membranes in serum‐free suspension but relocates to central apical membranes in cultures grown in Matrigel/serum.** (A) Immunolabelling for F‐actin (red) and ABCB1 (green), with DAPI (blue) in various serum‐free primary cultures. (B) Immunolabelling for F‐actin (red) and ABCB1 (green), with DAPI (blue) of C3953 and C105251 primary cultures grown as either serum‐free suspension colonies or as Matrigel‐embedded organoids in the presence of serum. Scale bars = 100 μm.

**Figure S4. Established colorectal cancer cell lines have polarised ABCB1.** (A) F‐actin/anti‐ABCB1 labelling of C80 colonies embedded in Matrigel with serum or grown as serum‐free suspensions. (B) Similar experiment to (A) but with the SW1222 cell line. Scale bars = 100 μm.

**Figure S5. C80 colonies grown in Matrigel/serum accumulate the ABCB1 substrate TMRE in lumens in an ABCB1‐dependent manner.** C80 colonies grown in Matrigel with serum or as serum‐free suspension cultures for 2 weeks and labelled with 100 μm ABCB1 substrate TMRE for 1 h, with or without ABCB1 inhibitors. Cultures were pre‐incubated with drug vehicle control (DMSO) or with the ABCB1 inhibitors verapamil (50 μm) or CP100356 (10 μm). Scale bars = 100 μm.

**Figure S6. ABCB1 distribution in colonies under different culture conditions.** (A) Mean ABCB1 fluorescence of 17 (from same image) C105251 colonies cultured as serum‐free suspensions or in Matrigel with serum for 1 week (*p* = 0.312 Student's *t*‐test). Error bars = SEM. (B) Anti‐ABCB1 labelling of collagen‐embedded C2284 colonies incubated with AIIB2 (β1 function blocking) or BIIG (isotype control) antibodies (10 μg/ml) for 1 week.

**Movie S1. Z‐stack of red F‐actin and blue DAPI staining of primary culture C2661 cultured in Matrigel with 10% serum for 1 week.** Steps are 5 μm.

**Movie S2. Three‐dimensional reconstruction of a C105251 monolayer grown on plastic in serum‐containing medium for 2 weeks, labelled for ABCB1 (green), F‐actin (red) and with DAPI (blue).** The scales show microns.

**Movie S3**. Primary cultures have collagen‐contracting capacity

**CEL files.** Four .CEL files each with raw microarray data from a cell line

**Table S1.** Microarray data used in Figures 2 and 4
