# Supplementary material for: Cellular polarity modulates drug resistance in primary colorectal cancers via orientation of the multidrug resistance protein ABCB1
Source: J Pathol. 2019 Jan 16;247(3):293–304. doi: 10.1002/path.5179 (PMC6519031; doi:10.1002/path.5179)
Supplement: Supplementary file 1 — Supplementary figure and movie legends [file PATH-247-293-s008.docx]

**Cellular polarity modulates drug resistance in primary colorectal cancers via orientation of the multidrug resistance protein ABCB1**

Ashley N *et al*. *J Pathol* 2018 (DOI: 10.1002/path.5179)

**Supplementary figure and movie legends**

**Figure S1. Immunostaining for polarity markers actin, ezrin, villin and myosin IIc, in six parental tumours and daughter primary serum-free suspension cultures.** (A) Haematoxylin/eosin staining of parental tumour sections. (B) Anti-actin (red)/anti-ezrin (green)/DAPI co-staining or (C) anti-villin (green)/anti-myosin IIC (red)/DAPI (blue) of parental tumour sections and corresponding daughter suspension cultures. (D) Anti-CEA labelling in green, DAPI in blue. Bar = 50 µm.

**Figure S2. Apical-in orientation of brush border proteins is restored in primary cultures grown in Matrigel with serum.** Primary suspension cultures were cultured as either serum-free suspensions or serum-containing Matrigel cultures for 1 week, followed by fixation, sectioning and immunolabelling. (A) Anti-actin (red)/DAPI (blue) and anti-ezrin (green) labelling of suspension/Matrigel cultures, with phase-contrast images. (B) Anti-myosin II (red)/DAPI (blue) and anti-villin (green) labelling.

**Figure S3. ABCB1 is polarised to outer colony cell membranes in serum-free suspension but relocates to central apical membranes in cultures grown in Matrigel/serum.** (A) Immunolabelling for F-actin (red) and ABCB1 (green), with DAPI (blue) in various serum-free primary cultures. (B) Immunolabelling for F-actin (red) and ABCB1 (green), with DAPI (blue) of C3953 and C105251 primary cultures grown as either serum-free suspension colonies or as Matrigel-embedded organoids in the presence of serum. Scale bars = 100 µm.

**Figure S4. Established colorectal cancer cell lines have polarised ABCB1.** (A) F-actin/anti-ABCB1 labelling of C80 colonies embedded in Matrigel with serum or grown as serum-free suspensions. (B) Similar experiment to (A) but with the SW1222 cell line. Scale bars = 100 µm.

**Figure S5. C80 colonies grown in Matrigel/serum accumulate the ABCB1 substrate TMRE in lumens in an ABCB1-dependent manner.** C80 colonies grown in Matrigel with serum or as serum-free suspension cultures for 2 weeks and labelled with 100 µm ABCB1 substrate TMRE for 1 h, with or without ABCB1 inhibitors. Cultures were pre-incubated with drug vehicle control (DMSO) or with the ABCB1 inhibitors verapamil (50 µm) or CP100356 (10 µm). Scale bars = 100 µm.

**Figure S6. ABCB1 distribution in colonies under different culture conditions.** (A) Mean ABCB1 fluorescence of 17 (from same image) C105251 colonies cultured as serum-free suspensions or in Matrigel with serum for 1 week (*p* = 0.312 Student’s *t*-test). Error bars = SEM. (B) Anti-ABCB1 labelling of collagen-embedded C2284 colonies incubated with AIIB2 (β1 function blocking) or BIIG (isotype control) antibodies (10 µg/ml) for 1 week.

**Movie S1. Z-stack of red F-actin and blue DAPI staining of primary culture C2661 cultured in Matrigel with 10% serum for 1 week.** Steps are 5 µm.

**Movie S2. Three-dimensional reconstruction of a C105251 monolayer grown on plastic in serum-containing medium for 2 weeks, labelled for ABCB1 (green), F-actin (red) and with DAPI (blue).** The scales show microns.
