# Supplementary figures and images for: Cellular polarity modulates drug resistance in primary colorectal cancers via orientation of the multidrug resistance protein ABCB1
Source: J Pathol. 2019 Jan 16;247(3):293–304. doi: 10.1002/path.5179 (PMC6519031; doi:10.1002/path.5179)

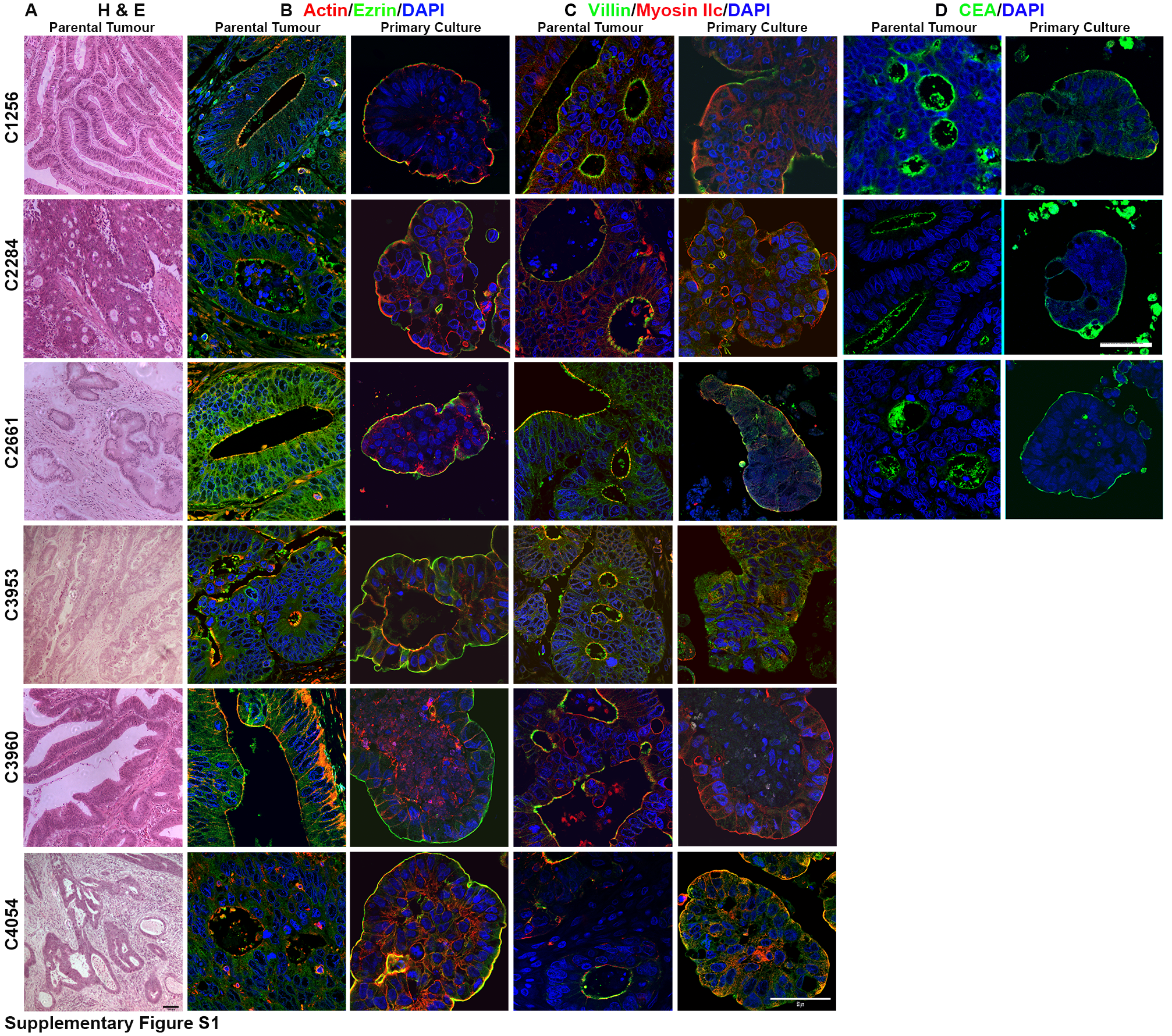

Supplement: Supplementary file 2 — Figure S1. Immunostaining for polarity markers actin, ezrin, villin and myosin IIc, in six parental tumours and daughter primary serum‐free suspension cultures. (A) Haematoxylin/eosin staining of parental tumour sections. (B) Anti‐actin (red)/anti‐ezrin (green)/DAPI co‐staining or (C) anti‐villin (green)/anti‐myosin IIC (red)/DAPI (blue) of parental tumour sections and corresponding daughter suspension cultures. (D) Anti‐CEA labelling in green, DAPI in blue. Bar = 50 μm. [file PATH-247-293-s005.tif]

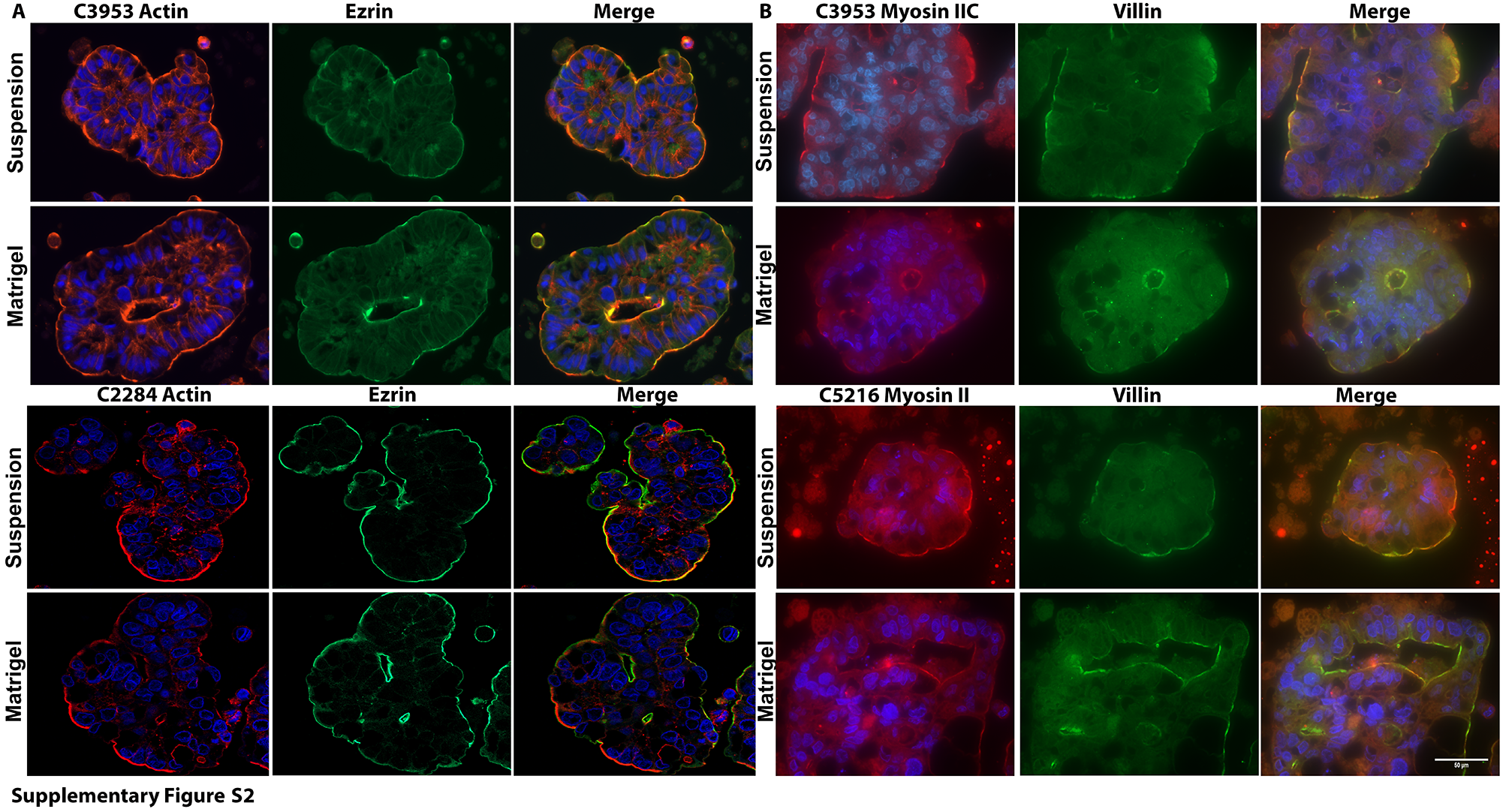

Supplement: Supplementary file 3 — Figure S2. Apical‐in orientation of brush border proteins is restored in primary cultures grown in Matrigel with serum. Primary suspension cultures were cultured as either serum‐free suspensions or serum‐containing Matrigel cultures for 1 week, followed by fixation, sectioning and immunolabelling. (A) Anti‐actin (red)/DAPI (blue) and anti‐ezrin (green) labelling of suspension/Matrigel cultures, with phase‐contrast images. (B) Anti‐myosin II (red)/DAPI (blue) and anti‐villin (green) labelling. [file PATH-247-293-s004.tif]

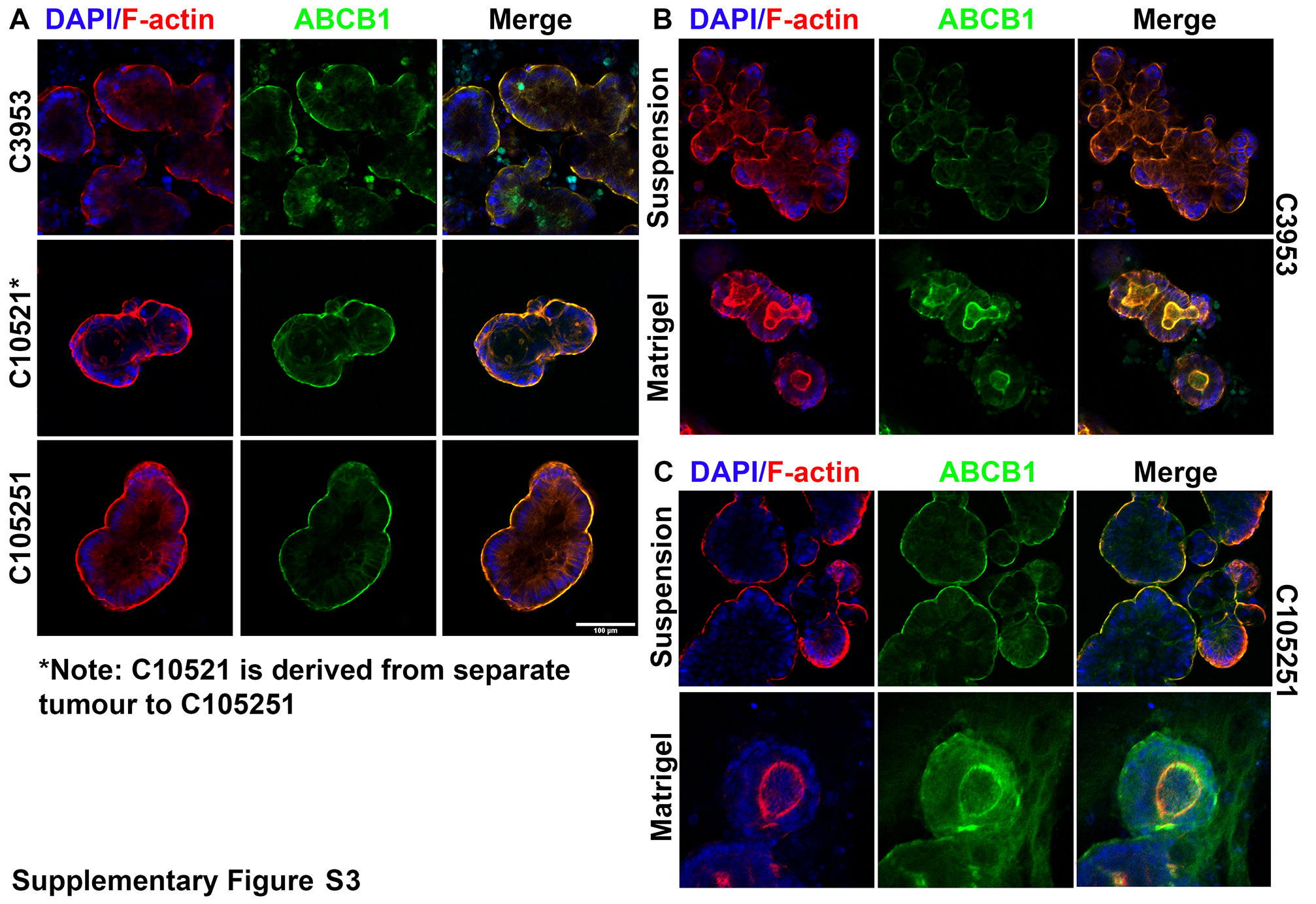

Supplement: Supplementary file 4 — Figure S3. ABCB1 is polarised to outer colony cell membranes in serum‐free suspension but relocates to central apical membranes in cultures grown in Matrigel/serum. (A) Immunolabelling for F‐actin (red) and ABCB1 (green), with DAPI (blue) in various serum‐free primary cultures. (B) Immunolabelling for F‐actin (red) and ABCB1 (green), with DAPI (blue) of C3953 and C105251 primary cultures grown as either serum‐free suspension colonies or as Matrigel‐embedded organoids in the presence of serum. Scale bars = 100 μm. [file PATH-247-293-s006.tif]

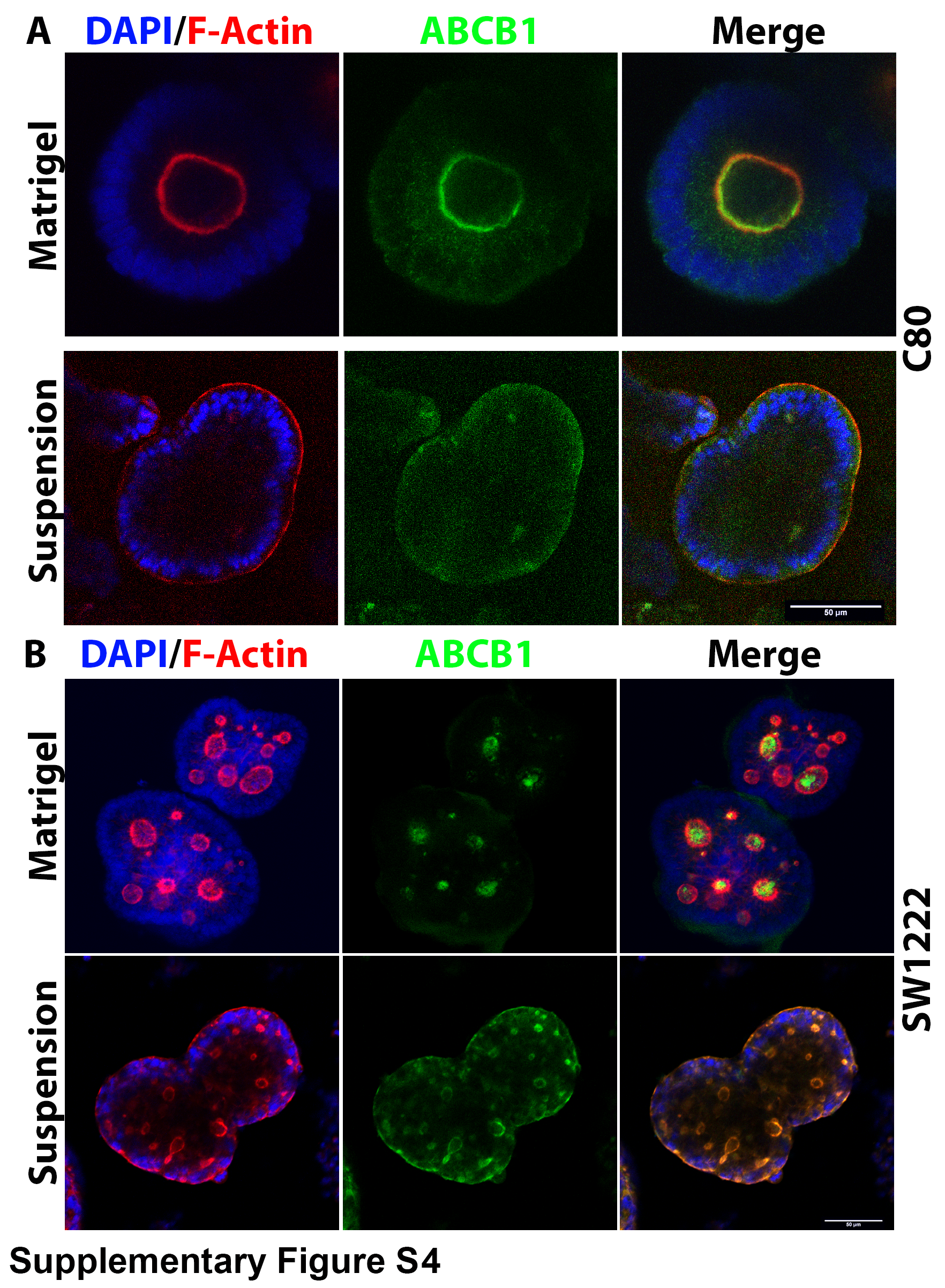

Supplement: Supplementary file 5 — Figure S4. Established colorectal cancer cell lines have polarised ABCB1. (A) F‐actin/anti‐ABCB1 labelling of C80 colonies embedded in Matrigel with serum or grown as serum‐free suspensions. (B) Similar experiment to (A) but with the SW1222 cell line. Scale bars = 100 μm. [file PATH-247-293-s001.tif]

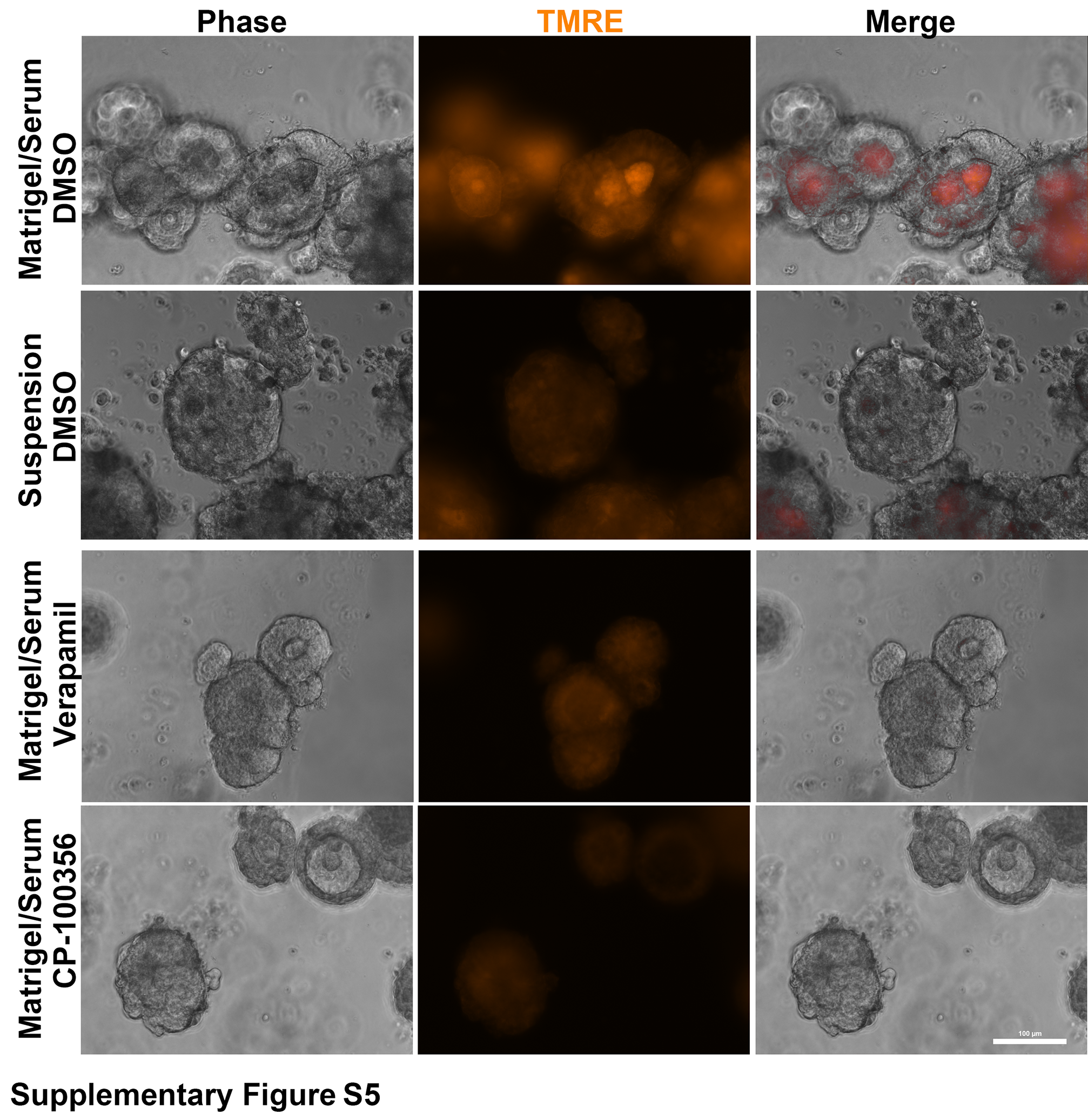

Supplement: Supplementary file 6 — Figure S5. C80 colonies grown in Matrigel/serum accumulate the ABCB1 substrate TMRE in lumens in an ABCB1‐dependent manner. C80 colonies grown in Matrigel with serum or as serum‐free suspension cultures for 2 weeks and labelled with 100 μm ABCB1 substrate TMRE for 1 h, with or without ABCB1 inhibitors. Cultures were pre‐incubated with drug vehicle control (DMSO) or with the ABCB1 inhibitors verapamil (50 μm) or CP100356 (10 μm). Scale bars = 100 μm. [file PATH-247-293-s012.tif]

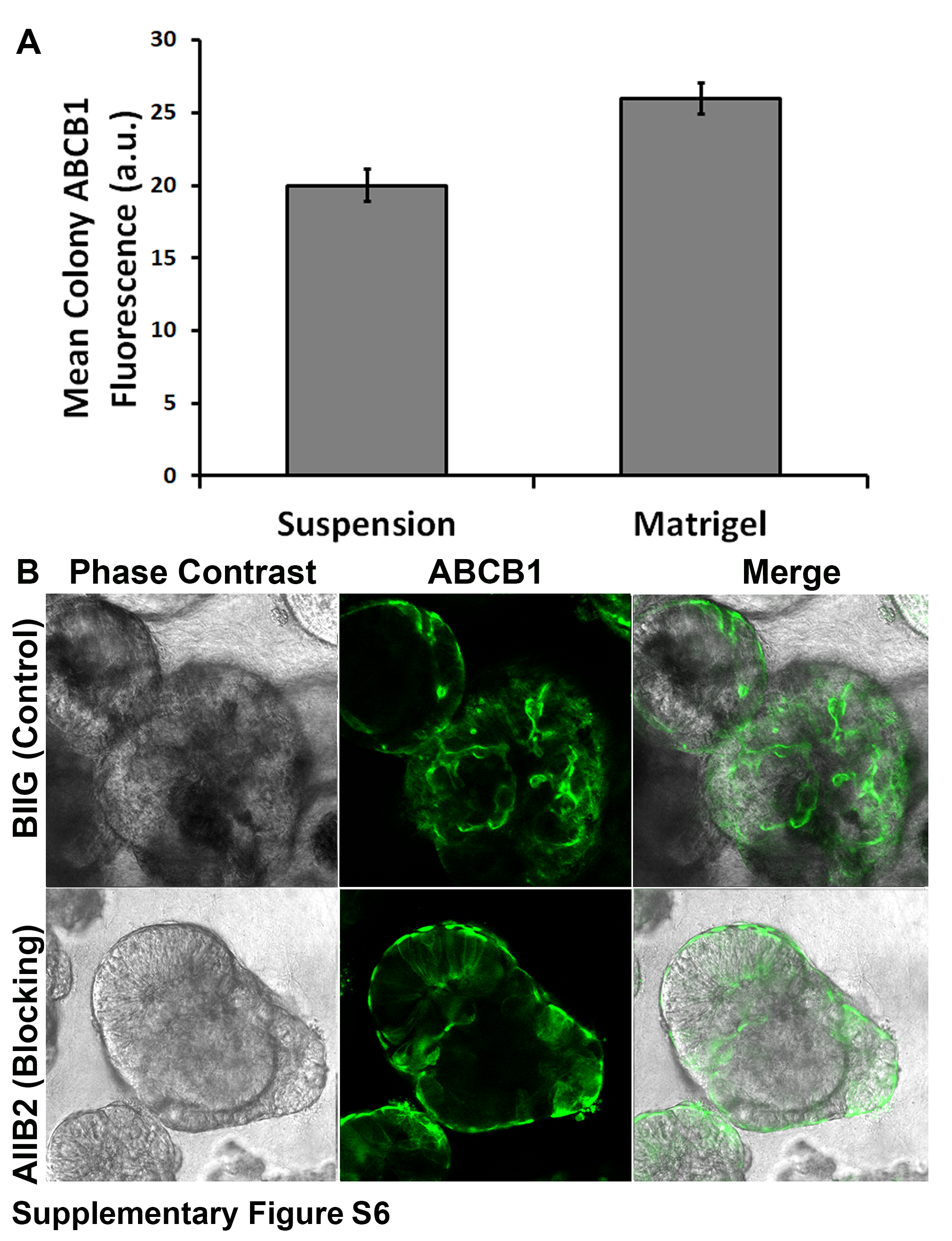

Supplement: Supplementary file 7 — Figure S6. ABCB1 distribution in colonies under different culture conditions. (A) Mean ABCB1 fluorescence of 17 (from same image) C105251 colonies cultured as serum‐free suspensions or in Matrigel with serum for 1 week (p = 0.312 Student's t‐test). Error bars = SEM. (B) Anti‐ABCB1 labelling of collagen‐embedded C2284 colonies incubated with AIIB2 (β1 function blocking) or BIIG (isotype control) antibodies (10 μg/ml) for 1 week. [file PATH-247-293-s002.tif]
